# The Reformed Public House

**Published:** 1924-02

**Authors:** A. W. Simons

**Affiliations:** 27 Meads Road, Wood Green, N. 22


### CORRESPONDENCE.

[Correspondence on all subjects is invited, but we cannot in any way be responsible for the opinions expressed by our correspondents. No communication can be entertained if the name and address of the correspondent are not given as a guarantee of good faith, but not necessarily for publication. All correspondents should write on one side of the paper only, and we cannot find space for very long letters.]

### The Reformed Public House.

Sir,—May I thank you for your admirably-written leaderette on this question. It exactly expresses the views of that large section of the public who are represented by no organisations—the moderate man. Messrs. Grubb and Dering appear to think that nothing can be done in this direction without calling in the Government to take over the licensed trade, and establish a monopoly. They apparently belong to the school of thought that would nationalise everything. They complain of a drink "trust," and want to substitute a "monopoly" for it!

If only the licensing justices would freely grant applications for improvements without, as in so many cases, imposing prohibitive restrictions, in a few years the public houses of the country would be revolutionised without the cost of a penny of the taxpayers' money. Hence the need for some measure on the lines of the Public House Improvement Bill. "To reform the public-house is a sounder and more hopeful aim than the policy of prohibition or even mere reduction. It is well known that the virtue of self-respect is imperceptibly reinforced in an atmosphere of wholesome comfort."

A. W. Simons.

27 Meads Road,
Wood Green, N. 22.

